# Patient Perspectives on Strengths and Challenges of Therapist-Assisted Internet-Delivered Cognitive Behaviour Therapy: Using the Patient Voice to Improve Care

**DOI:** 10.1007/s10597-018-0286-0

**Published:** 2018-05-26

**Authors:** H. D. Hadjistavropoulos, Y. N. Faller, A. Klatt, M. N. Nugent, B. F. Dear, N. Titov

**Affiliations:** 1 Department of Psychology, University of Regina, 3737 Wascana Parkway, Regina, SK S4S 0A2 Canada; 2 Department of Psychology and eCentreClinic, Macquarie University, Sydney, NSW 2109 Australia; 3 Department of Psychology, MindSpot Clinic, and eCentreClinic, Macquarie University, Sydney, NSW 2109 Australia

**Keywords:** Patient-centered, Internet, Cognitive behaviour therapy, Depression, Anxiety, Therapist-assisted

## Abstract

Therapist-assisted internet-delivered cognitive behaviour therapy (T-ICBT) involves patients reading online treatment materials, completing relevant exercises, and receiving therapist support. This study aimed to understand the preferences and recommendations of 225 patients enrolled in a T-ICBT course for depression and anxiety via an online therapy unit in collaboration with community mental health clinics dispersed across one Canadian province. An open-ended survey asked participants their opinions of the course and responses were analyzed using a content analysis approach. Patient comments addressed many strengths of the course (64%), with some opportunities for improvement (36%). Most-appreciated features included ability to download content for future use, reading other patients’ experiences, and content of lessons. Patients made suggestions for improving the breadth of patient stories, timeline of the course, and matching availability of the therapist to patient need. Patient feedback regarding preferences provides valuable information for improving the patient-centered nature of T-ICBT.

## Introduction

Depression and anxiety are prevalent and disabling conditions (van Belijouw et al. 2010). It is estimated, however, that approximately 40% of individuals requiring services for these disorders do not receive formal treatments such as cognitive behaviour therapy (CBT) (Urbanoski et al. 2017). CBT is a psychological treatment that helps individuals identify and manage unhelpful thoughts and behaviours, and is known to be an efficacious treatment (Hollon et al. 2006). Factors such as time or mobility constraints, rural or remote location, limited access to specialized providers as well as disability and stigma can interfere with accessing CBT (Shafran et al. 2009). Therapist-assisted internet-delivered CBT (T-ICBT) has great potential to improve patient access to CBT (Andersson and Titov 2014). In T-ICBT, patients review written materials on a weekly basis that contain the same information and skills as those taught in face-to-face CBT. In addition, patients have access to a therapist via weekly emails or phone calls.

There is growing research on the effectiveness of T-ICBT for depression and anxiety with randomized controlled trials demonstrating that those who receive T-ICBT improve to a greater degree than those in control conditions (Andersson et al. 2014; Hedman et al. 2012). Importantly, T-ICBT outcomes are similar to outcomes for face-to-face therapy (Andersson et al. 2013) and several studies have found that effects are maintained longer term (Andersson et al. 2013; Eysenbach et al. 2011; Paxling et al. 2011). T-ICBT is clearly a promising approach to increase community access to patient-centered care, which refers to “providing care that is respectful of and responsive to individual patient preferences, needs, and values” (Institute of Medicine Committee on Quality of Health Care in America 2001, p. 6).

While the research evidence is promising, T-ICBT is largely unavailable on a routine basis for most patients (Hadjistavropoulos et al. 2017). At this point, we do not fully understand what aspects of treatment are most valued and perceived as beneficial, which could provide helpful information to others who are interested in offering T-ICBT. Qualitative research is well suited for exploring patient perspectives on T-ICBT and identifying methods to improve patient-centered care. In one previous study, Richards et al. (2016) examined users’ (*n* = 281) experiences with an 8-week supported ICBT program for depression. The authors found that ICBT users were satisfied with ICBT for depression, and that this was largely due to the accessibility and time flexibility of the online environment. This study also found that patients desired more personalization and guidance. Another study, by Lillevoll et al. (2013), used a small sample (*n* = 14), and a phenomenological-hermeneutical approach to discern which dimensions of ICBT for depression patients found helpful. Three components were important to patients: having a source of knowledge (regarding depression), using that knowledge personally, and engaging in therapist interaction. Respondents’ negative experiences with ICBT and strategies for improving ICBT were not investigated.

The current study contributes to the literature by examining patient feedback on a transdiagnostic T-ICBT program offered via an Online Therapy Unit specifically funded to support patients in the community in accessing care for depression and anxiety. In transdiagnostic T-ICBT, patients are provided with treatment materials that assist with symptoms of depression and/or anxiety (Dear et al. 2015; Titov et al. 2014, 2015). Similar to, Richards et al. (2016), the current study inquired about both what was liked and disliked about T-ICBT, to understand what particular features should be retained and which could be improved for more patient-centered care. Information from this study (a) ensures that helpful components of T-ICBT are highlighted for other clinicians who have an interest in delivering this treatment, and (b) identifies opportunities to improve T-ICBT by incorporating patient feedback into the delivery of any T-ICBT program.

## Methods

### Context, Participants, Recruitment, and Eligibility Criteria

The Online Therapy Unit, funded by the provincial government, is responsible for the delivery of T-ICBT to Saskatchewan residents. To access T-ICBT, patients complete an online screening questionnaire that asks patients about demographics and symptom severity (via Patient Health Questionnaire-9 and Generalized Anxiety Disorder-7 questionnaires). Patients who identify in the online questionnaires that they are under the age of 18 years, are not a Saskatchewan resident, and do not have access to a computer and/or the internet are immediately excluded from T-ICBT. Following the online screen, a telephone call is used to further assess eligibility for T-ICBT. Patients are referred for face-to-face services if they identify as being at high risk of suicide, or have significant problems with alcohol or drugs, or psychotic disorders. If appropriate for T-ICBT, patients are given a username and password to access the T-ICBT course (described below), which is hosted on a secure web application developed for the Unit. Patients are assigned to a designated therapist for email or telephone contact over 8 weeks. These therapists work in community mental health clinics (38%) or directly in the Online Therapy Unit (62%) and are given login credentials to access the secure web application as well.

Between September 2015 and the end of May 2016, 225 people experiencing depression and/or anxiety began T-ICBT. Patients learned of the course from family members or friends (*n* = 28; 12%), advertisements (*n* = 37; 17%), health care providers (*n* = 81; 36%), or mental health professionals (*n* = 79; 35%). Prior to analysis, and to further protect privacy, patient names were removed from the dataset, and replaced with case identification numbers. On average, participants were middle-aged (*M*_age_ = 37.8; *SD* = 14.6) and the majority were female (*n* = 158; 70%). Most were Caucasian (*n* = 203; 90%), married (*n* = 131; 58%), living in an urban setting of more than 10,000 people (*n* = 149; 66%), had completed at least some post-secondary education (*n* = 168; 75%), and were employed (*n* = 144; 64%). Over half of the participants were on medication for depression or anxiety (*n* = 143; 64%). Over half (*n* = 135; 60%) answered open-ended questions regarding their preferences and recommendations for the course at the end of treatment; these qualitative responses comprised the data for this study.

### T-ICBT Course for Depression & Anxiety

The transdiagnostic T-ICBT program, known as the *Wellbeing Course*, was developed by a research team at the Macquarie University in Sydney, Australia and has been found effective at addressing both depression and anxiety in Australia and Canada (Hadjistavropoulos et al. 2016; Titov et al. 2015). Patients gradually worked on five psychoeducational lessons over eight weeks. All lessons were written for a Grade 6 reading level and addressed (a) symptom identification and the cognitive behavioural model; (b) thought monitoring and challenging; (c) de-arousal strategies and pleasant activity scheduling; (d) graduated exposure; and (e) relapse prevention (Titov et al. 2015). The lesson materials include textual and visual slideshows, and additional resource materials. Patients also review vignettes about two characters, Jo and Glenn, who were former patients of the T-ICBT course, and whose thought processes and behaviours are shared with current patients to facilitate a link between the psychoeducation and situations in everyday life. At the end of each lesson, patients are presented with a downloadable do-it-yourself (DIY) guide that summarizes lesson materials and recommends homework to patients. Along with their self-guided work, patients were matched with a therapist (Hadjistavropoulos et al. 2016). This therapist reviewed symptom measures that patients completed prior to each lesson and at post-treatment. On a weekly basis, the therapist responded to patient emails sent to the therapist during the preceding week. Some phone contact was also provided if a patient had not logged on during the week or if the patient requested a telephone conversation with the therapist.

### Measures

The Post Treatment Questionnaire included two open-ended questions that asked patients: (a) what they liked about the course; and (b) what they did not like about the course and should be improved.

### Data Analysis

Survey responses were collected over a secure server and saved in an SPSS document. Data was analyzed for themes using a conventional content analysis approach (Hsieh and Shannon 2005), and NVivo qualitative software. This approach was chosen since there were minimal theories and research conducted on patient’s perspectives of T-ICBT. The data was explored by applying qualitative methods of coding and grouping codes into themes, as well as quantitative methods of counting, and calculating descriptive statistics (Creswell 2011; Hsieh and Shannon 2005). First, each response was read closely, and researchers met to discuss initial impressions and created a coding guide of key words and definitions. The data was reviewed again and respondents’ comments were then coded using the newly adopted coding guide. Two coders worked independently, and met frequently to discuss discrepancies, fine-tune code descriptions, and to reach consensus on coding. Finally, the data was approached by an expert coder (HH) who confirmed categories and compared the coded data for overlooked themes. The expert coder was also available to resolve any discrepancies in coding between the two primary coders. To ensure rigor and transparency, reflective memoing was employed during data analysis (Birks et al. 2008).

### Compliance with Ethical Standards

This study was approved by the University of Regina Research Ethics Board. Informed consent was obtained from all individual participants included in the study. The authors certify that they accept responsibility for the conduct of the study, and for the analysis and interpretation of the data. The authors helped write the manuscript, agree with the decisions made, and have reviewed and approved the final manuscript. The authors meet the definition of an author as stated by the International Committee of Medical Journal Editors.

## Results

### Overview

Of the 135 survey respondents, on average, respondents made 2.05 (*SD* = 1.04) separate comments, resulting in coding of 278 comments. Of the 278 comments, 45% (*n* = 124/278) were about the cognitive behavioural information taught during the course (content); 37% (*n* = 104/278) related to the patient’s experience of learning and practicing the skills of the course (process); and 18% (*n* = 50/278) included feedback regarding communication with the assigned therapist (therapist contact). Each domain is described in greater detail below.

### Content

Of the 124 comments related to program content, 70% (*n* = 87/124) were complimentary, while 30% (*n* = 37/124) were opportunities for change. Many of the comments in this category (*n* = 70/124; 56%) were about two characters whose stories were shared with patients to demonstrate how the skills can be applied. Most comments about the stories (*n* = 49/70), reflected that that stories were a strength of the program: “The stories are a good way to help relate the more theoretical material to real life situations.” Nevertheless, other story-related comments (*n* = 21/70) reflected that some participants felt unable to relate to the stories, finding them boring or artificial representations of what it is like to struggle with depression and anxiety. This is reflected in the following patient comment “I did not relate to well to the situations and symptoms of the two examples.”

Another 28% (*n* = 35/124) of comments were about the content of lessons generally, including the design, organization, or length of the T-ICBT lessons. The majority of these comments (*n* = 24/35) reflected positive comments about the lessons as being informative, clear, well laid-out, or easy to follow. The remaining lesson comments (*n* = 11/35) provided suggestions for improvement, with patients articulating their preference for “more in-depth information,” or less of a “scripted and repetitive” nature of lessons. Patients’ ideas for improving the materials included adding in more examples, adding audio and/or visual components, adding more information, or by contrast, reducing information.

Finally, 15% (*n* = 19/124) of patients’ comments about the content of the course were about additional resources (materials that can be downloaded and used to augment the primary knowledge in the lessons). The majority of comments (*n* = 14/19) highlighted patient enjoyment of specific materials (i.e. resources on sleep problems, and assertiveness), although some comments (*n* = 5/19) described a need for additional resources beyond those available, on topics such as emotions, time management, and how to cope with panic.

### Process

Comments describing the patient’s experience with T-ICBT content were deemed process comments and accounted for 37% (*n* = 104/278) of all coded comments. Broadly, patients reported their experiences related to process as 72% (*n* = 75/104) strengths and 28% (*n* = 29/104) opportunities for improvement.

Many of the process comments, 63% (*n* = 66/104) concerned the printable DIY summaries of lessons. Most patients (*n* = 59/66) reported finding the process of being able to download and work on homework assignments suggested in the DIY materials as beneficial and felt it was helpful to have this material available for longer term access. Nevertheless, some comments (*n* = 7/66), reflected dissatisfaction that the DIY guides were “lengthy repeats of slideshow” materials.

Approximately 15% (*n* = 16/104) of patient’ comments about process were about the course timeline. The majority of patients’ comments (*n* = 13/16) noted opportunities for change, while very few (*n* = 3/16) expressed satisfaction with the timeline. Most often participants reported needing “more time to practice skills between lessons.” Reasons varied from being busy and feeling rushed, to wanting to focus on developing one skill before moving on. Others noticed that when they were suffering from depressive symptoms, they devoted less time to their lessons, and therefore would have liked more time to work through the lessons.

The online format was mentioned in 14% (*n* = 15/104) of patient comments about their therapy process. Most comments (*n* = 13/15) reflected an appreciation for therapy being delivered online, and with material being available anytime for them to work on at their convenience. Nevertheless, there were a few comments (*n* = 2/15) to suggest some dissatisfaction with the online format. One patient felt constricted by the online delivery and another patient highlighted how online therapy required greater “self-motivation to get through the lessons”.

Though mentioned infrequently, 6% of process comments (*n* = 7/104) were about the symptom questionnaires patients completed at the beginning of each lesson and post-treatment. Specifically, patients reported having difficulty distinguishing between whether symptoms were present “Not at all” or “Several Days” indicating that this was “too big of a jump” between the options.

### Therapist Contact

In this study, 18% (*n* = 50/278) of all patients’ comments concerned therapist contact. Comments in the therapist category represented 30% (*n* = 15/50) strengths and 70% (*n* = 35/50) opportunities for change.

Within this category, 46% (*n* = 23/50) of the therapist contact comments were about the nature of the contact, that is, the patient’s impression of the quality and overall sense of their therapeutic relationship. Patients had mixed perceptions of the therapist contact. Among the comments noting strengths (*n* = 13/23), patients expressed the value of having a trained professional to communicate with on a weekly basis. One comment noted that the strength of T-ICBT is its blend of information and communication “I liked the mix of lessons and talking to a therapist. Instead of this course just being a place to vent to a therapist it was a place that gave me tools”.

Most of the comments within nature of contact were about appreciating consultation, access, support, and encouragement. For some patients, however, comments reflected (*n* = 10/23) confusion about the role of the therapist in this treatment format, as well as a yearning for more connection and personal quality to the emails that were exchanged between patient and therapist. Patients asked for “more personal conversation,” and disclosed that “I found the emailed responses somewhat “canned” (as though you copy and paste and fill in the blank with some information from my emails).” Generally, opportunities for change were about more direction, depth, and support from the therapist for the patient.

Approximately 28% (*n* = 14/50) of comments about the therapist focused on the type of contact, such as email, telephone, or in-person. The majority of comments (*n* = 12/14) suggested that patients wanted improvements to the therapist contact. Mostly, patients expressed a desire for more personal contact, via phone calls, with their therapists: “Maybe more voice conversations;” “I think it would be nice to have a few phone calls included with the emailing.”

Finally, 26% (*n* = 13/50) of comments made about therapists concerned the availability of the therapist. All of the comments represented opportunities for change, suggesting that patients’ experiences of T-ICBT would be improved if patients and therapists had more interactions throughout the course. This is illustrated by comments such as “I think I would have liked to receive more email communication…” and “Have someone available a little more often. Once a week is kind of a long time for a short course.”

### Overall Strengths and Opportunities for Improvement

Overall, 64% (*n* = 177/278) of all patient comments represented strengths, while 36% (*n* = 101/278) provided opportunities to improve the patient’s experience of T-ICBT. The most common strengths mentioned across all areas included having DIY downloadable guides (*n* = 59/177; 33%), inclusion of stories (*n* = 49/177; 28%), and cognitive behavioural materials (*n* = 24/177; 14%). The most common areas identified as needing improvement included the stories (*n* = 21/101; 21%), the timeline (*n* = 13/101; 13%), and therapist availability (*n* = 13/101; 13%).

## Discussion

This study aimed to understand patients’ experiences of T-ICBT by focusing on their preferences, as represented in post-treatment questionnaires. Although the findings are specific to the T-ICBT program used in this study, they provide valuable information for other clinicians who have an interest in developing and offering T-ICBT to improve access to mental health care. A strength of the study is that 60% of those who participated in T-ICBT provided feedback, with an average of 2.05 comments per respondent. The findings specifically highlight features of T-ICBT programs, processes and therapist-assistance that were most appreciated as well as features that presented challenges to patients. The findings are aligned with Richards et al. (2016) high satisfaction ratings and Lillevoll et al. (2013) critical categories, and extend knowledge of patient preferences related to T-ICBT. The study results could shape development of T-ICBT interventions for depression and anxiety going forward.

Main findings are that the content component of transdiagnostic T-ICBT seems highly acceptable. Participants commented about this the most, and most of these comments indicate that patients appreciate what they are learning and how information is presented. Overwhelmingly, patients like learning cognitive and behavioural skills via stories in the lessons to cope with their symptoms of both anxiety and depression. This suggests that presentation of core cognitive behavioural content should be considered essential to T-ICBT programs. The inclusion of narratives in teaching materials has been well-researched and should be acknowledged as a factor that many individuals found beneficial (Hinyard and Kreuter 2007; Mar 2004). It must be noted, however, that a portion of participants did not find value in the stories as presently offered. This divergence in patient opinions indicates that future research could be dedicated towards examining the impact of varying storylines on patient outcomes.

Generally, fewer comments were made about the process of therapy. What was said made it clear that patients value having downloadable content to retain and use beyond treatment completion. There were also some suggestions for patients to be able to adjust their treatment timelines to meet their learning needs, which is in line with past research (Richards et al. 2016; Rozental et al. 2015).

There were also very few comments about therapist contact. Perhaps this reflects that therapist support is a small component of patients’ experiences of T-ICBT. When comments were made, they varied widely, and exemplified mixed feelings about the nature of therapist contact. Some patients perceived the current nature of therapist-assistance to be the level of support they wanted while others desired more support by email and or by phone. An implication, consistent with the literature is that there may be benefit in tailoring therapist support to patient needs (Lillevoll et al. 2013; Richards et al. 2016; Rozental et al. 2015). This patient feedback parallels recent therapist feedback whereby therapists also expressed a desire to tailor the amount and nature of support to patients (Hadjistavropoulos et al. 2017).

In terms of limitations, this study examined feedback from questionnaire completers (60% response rate). Patients who did not complete the T-ICBT course could have provided vital information to understanding potential improvements to ICBT. That noted, the value of obtaining feedback from program completers should not be discounted as it provides valuable information for others who have an interest in developing or offering T-ICBT. Examining feedback from the current sample in particular is helpful given that overall the T-ICBT program used for this study has been found efficacious (Hadjistavropoulos et al. 2016; Titov et al. 2015). A related limitation is that a great deal of the coded comments were single terms or concepts. Researchers worked with fragments of information to understand patient preferences. It is likely that deeper understanding of patient preferences would be achieved by conducting interviews. Interviews, however, do not lend themselves to understanding the frequency of comments and are typically conducted on a much smaller sample. It should also be noted that T-ICBT in this research largely involved patients sending written secure messages. T-ICBT, however, can involve other methods of communication (e.g., greater phone calls, chat-based interaction) and feedback from patients could be different with different methods of ICBT treatment provision.

## Implications

As the field of T-ICBT emerges to improve patient access to treatment, providers are well-placed to consider patient perspectives in the development of treatment programs. This study examined patient feedback on T-ICBT for depression and anxiety. Few other projects, to date, have taken a qualitative approach to understanding the treatment-user’s experience. The findings of this study show strong preference for learning cognitive and behavioural skills, especially by reading case-based stories and using the accompanying guides that can be retained longer term. For improved patient-centered care, enhancement of patient stories, to reflect more situations of depression and anxiety, would be valuable. Since personalized timelines and availability of the therapist represent opportunities for development of T-ICBT, future research could explore how more tailored contact could support patients’ wellbeing. These insights can inform other care providers, as they reach to offer accessible treatment options to a growing population of patients. Hopefully, as research incorporates the voices and knowledge of those receiving mental health treatment, T-ICBT will be shaped to deliver even better care.
